# Analysis of YouTube videos on rheumatic diseases and pregnancy: a cross-sectional study

**DOI:** 10.1007/s00296-026-06102-7

**Published:** 2026-03-28

**Authors:** Dinara Yerlanova, Olena Zimba, Umida Khojakulova, Marlen Yessirkepov, Burhan Fatih Kocyigit

**Affiliations:** 1 Department of Chemical Disciplines, Biology and Biochemistry, South Kazakhstan Medical Academy, Shymkent, Kazakhstan; 2 Department of Rheumatology, Immunology and Internal Medicine, University Hospital in Kraków, Kraków, Poland; 3 National Institute of Geriatrics, Rheumatology and Rehabilitation, Warsaw, Poland; 4 Department of Internal Medicine N2, Danylo Halytsky Lviv National Medical University, Lviv, Ukraine; 5 Department of Emergency Medicine and Nursing, South Kazakhstan Medical Academy, Shymkent, Kazakhstan; 6 Center for Life and Health Sciences, National Academy of Sciences, Under the President of the Republic of Kazakhstan, Almaty, Kazakhstan; 7 Department of Physical Medicine and Rehabilitation, University of Health Sciences, Adana City Research and Training Hospital, Adana, Türkiye

**Keywords:** Rheumatic diseases, Rheumatology, Social media, Information science, Pregnacy

## Abstract

This study assessed the quality and reliability of YouTube videos on rheumatic diseases and pregnancy, analyzing video sources, presentation formats, user interaction metrics, and common topics. In this cross-sectional study, YouTube searches were conducted on February 2, 2026, using the keywords “rheumatology pregnancy”, “rheumatic diseases pregnancy”, “rheumatism pregnancy”, and “arthritis pregnancy”. The top 50 results for each keyword were evaluated, yielding a total of 200 videos. For each video, the number of views, likes, comments, and the number of days since upload were recorded; daily data were calculated. Videos were classified by presentation method. Using a classification scheme developed for the thematic distribution of video content, all topic categories in each video were recorded. Videos were categorized into low, intermediate, and high quality groups based on their Global Quality Scale (GQS) scores. After applying exclusion criteria, 55 videos were included in the analysis. Of these, 15 (27.3%) were classified as low quality, 16 (29.1%) as intermediate, and 24 (43.6%) as high quality. The most common presentation format was narrative-focused (*n* = 35, 63.6%). All videos presented as panel/webinar (*n* = 5, 100%) were rated high quality. Daily views (*p* = 0.285) and daily comment counts (*p* = 0.978) did not differ between quality groups, whereas daily likes were higher in the low quality group (*p* = 0.014). The most frequently discussed topics were the safety of anti-rheumatic drugs (*n* = 28), pre-pregnancy family planning (*n* = 27), management of disease activity during pregnancy (*n* = 21), and pregnancy outcomes in rheumatic diseases (*n* = 17). The quality of content on rheumatic diseases and pregnancy on YouTube is heterogeneous. User engagement metrics do not reliably reflect content quality. Increasing the production of structured, guideline-based contents, supporting materials to correct misinformation, and evaluating different platforms will improve digital health communication in the field of rheumatic diseases and pregnancy.

## Introductıon

Rheumatic diseases are chronic inflammatory conditions mostly affecting women of reproductive age, resulting in clinical implications during pregnancy that impact maternal and fetal health [1]. Rheumatoid arthritis, systemic lupus erythematosus, antiphospholipid syndrome, and other connective tissue disorders require a meticulous, multidisciplinary approach to pregnancy planning, disease activity management, drug safety, and postpartum care [2, 3, 4]. The fluctuations in disease activity throughout pregnancy, the heightened risk of flares in certain cases, and the potential adverse effects of anti-rheumatic drugs on the fetus complicate the course of pregnancy in this patient population. Consequently, precise scheduling, appropriate management, and evidence-based information are essential for safe pregnancy care in women with rheumatic diseases [5, 6].

In recent years, a notable transformation has occurred in consumers’ behavior regarding the acquisition of health information, with internet-based resources emerging as a crucial source of information [7]. Video-sharing platforms, particularly YouTube, are widely used to obtain health information due to their accessibility and visual features. YouTube’s free, globally accessible platform empowers consumers to access health-related contents without temporal or geographic limitations [8, 9]. Nonetheless, the scientific accuracy, reliability, and educational quality of the content posted on these platforms lack consistent oversight. This situation may foster an environment conducive to the spread of misinformation, especially on delicate subjects like pregnancy and chronic disease management. Assessing the quality and reliability of online contents on clinically complex topics, such as rheumatic diseases and pregnancy, is essential for patient education and public health [10].

This study aimed to thoroughly assess the content quality and reliability of YouTube videos regarding rheumatic diseases and pregnancy. Videos were categorized into low, intermediate, and high quality based on their quality scores. Additionally, relationships between quality levels and the source types and presentation formats of the videos were examined. User engagement metrics were compared across the quality groups, and the most commonly addressed topics in the videos were identified.

## Methods

This study was planned using a cross-sectional design. Video scanning was performed on February 2, 2026. The study involved searching on YouTube using the keywords “rheumatology pregnancy”, “rheumatic diseases pregnancy”, “rheumatism pregnancy”, and “arthritis pregnancy”. Because YouTube provides personalized results based on user behavior, searches were performed in incognito mode after clearing browser cookies and browsing history to minimize personalization bias and obtain standardized results. This approach aimed to reduce the influence of user-specific algorithmic guidance, thereby providing a more objective video selection.

Search results were obtained using the “Relevance” option that mirrors the standard user experience and constitutes the platform’s default ranking configuration [11, 12]. Prior research indicates that online users often scrutinize only a limited subset of search results and predominantly engage with information at higher ranks/traffic. Consequently, the first 50 videos for each search keyword were assessed to accurately represent actual user activity [13, 14]. On the stated date, 200 video URLs were documented, and key video parameters were collected simultaneously.

The exclusion criteria were established in advance. Videos in languages other than English, duplicate materials, unrelated videos, and content with substantial technical faults in audio or image quality were excluded from the analysis. Furthermore, videos under one minute were excluded from the analysis because they were considered insufficient to convey substantive information.

During the video assessment procedure, two independent researchers conducted separate evaluations of the videos, remaining unaware of each other’s scores. Following the initial assessments, the researchers’ scores were compared, and videos with divergent ratings were identified. Videos exhibiting inconsistencies in the evaluation results were subsequently reviewed by a third researcher, who determined the final decision. The concordance between the researchers’ evaluations was quantified using Cohen’s kappa coefficient, thereby indicating the reliability of the evaluation process [15].

### Video parameters

Video metrics include total views, likes, and comments; the video’s length in seconds; and the number of days between the upload date and the access date. Daily engagement metrics (daily views, daily likes, and daily comments) are computed by dividing the cumulative totals by the number of days since the video’s upload date. Furthermore, videos are classified into five established categories based on their content presentation format: (a) Narrative-oriented videos feature speaker-focused content, typically with few visual aids, in which a physician or healthcare professional communicates directly to the camera, conveying information verbally; these films are generally presented in a “doctor speaking” manner, with some organized as question-and-answer sessions; (b) Slide-based presentations convey information through visual elements, such as PowerPoint; they may include bullet points, tables, diagrams, guideline summaries, and statistical data, and are frequently scholarly in character; (c) Animated visual explanatory videos elucidate intricate clinical procedures through animation, graphics, diagrams, or visual metaphors; this category encompasses visually intense contents, including animations depicting fetal development, schematics illustrating the immune system, risk ratio presentations accompanied by graphs, and animated infographics; (d) Patient experience-based videos feature unique experiences, typically conveyed in a patient-centered, personal narrative format; in such materials, therapeutic contents may be subordinate, with the story primarily rooted in an emotional or personal perspective; (e) The panel-webinar format often consists of multiple speakers, discussions, or question-and-answer sessions and is offered as expert panels, moderated webinars, or conference recordings; this category often encompasses organized presentations that highlight multidisciplinary viewpoints.

### Sources

Videos are categorized by source, the content developer’s professional identity, and the institutional framework to which they are affiliated. The physician category encompasses videos that are either directly created by a specialized physician or formally published under a physician’s name. The nonphysician healthcare professional category encompasses materials created by nurses, physiotherapists, dietitians, or other healthcare practitioners. The category of academic medical centers encompasses videos generated within university hospitals or academic healthcare facilities. Non-academic healthcare facilities encompass materials disseminated by private hospitals, clinics, or health centers. TV channels or news media category covers content delivered through health-themed programs or news formats; the nonprofit charities or foundationscategory comprises content from civil society organizations involved in patient advocacy, awareness, and education; the pharmaceutical or commercial company category refers to content disseminated by pharmaceutical firms or commercial healthcare entities; and the independent user category consists of videos uploaded by individual content creators whose corporate affiliation is not clearly identified.

### Content assessment

A prominent method for assessing online educational materials is the Global Quality Scale (GQS). The GQS is designed to assess the informativeness and educational value of digital health materials on a five-point scale (1–5). A score of 1 indicates that the material is deficient, inconsistent, contains substantial information gaps, and offers minimal value to the user; a score of 5 denotes that the content is exceptionally consistent, complete, and highly beneficial from an educational standpoint [16].

In this study, videos were assessed with their GQS scores and grouped into quality categories. Those with scores of 4 or 5 were labeled “high quality”, 3 “intermediate quality”, and 1 or 2 “low quality.” This approach, which uses GQS categories, has been favored in previous studies because it provides a more objective and comparable means of evaluating content quality [17].

To evaluate the reliability of video content, a modified DISCERN tool, commonly used to assess online health and educational materials, was employed. This tool systematically reviews criteria, including the clarity and comprehensibility of information, content neutrality, potential for bias, and the use of references and supplementary data. The scale comprises five questions, answered ‘yes’ or ‘no’; each positive answer is worth 1 point, and each negative answer is worth 0 point. The total score ranges from 0 to 5, with higher scores indicating greater reliability of the video information [18].

### Topic assessment

A topic classification approach tailored to rheumatic diseases and pregnancy was developed to assess the distribution of themes in video content. Given that videos may encompass multiple topics, each video was meticulously analyzed, and all topics discussed were recorded. The topic classification was organized into nine main categories. The first category is *‘Preconception - Family Planning*,’ which encompasses topics including pregnancy timing, contraceptive advice, pre-pregnancy counseling, and the importance of a multidisciplinary approach. The second category centers on *‘Disease Activity - Management During Pregnancy*’ assessing how disease activity progresses during pregnancy, the risk of flare-ups, management strategies for each trimester, and how pregnancy influences the disease. The third category addresses the *‘Safety of Anti-Rheumatic Drugs’* and reviews conventional DMARDs, biologics, corticosteroids, nonsteroidal anti-inflammatory drugs, and medications that should be discontinued before pregnancy. The fourth category covers *‘Pregnancy Outcomes in Rheumatic Diseases’* which includes maternal and fetal outcomes, preterm birth, miscarriage or stillbirth, and mode of delivery. The fifth category addresses *‘Disease - Specific Considerations’*; pregnancy management is evaluated in the context of systemic lupus erythematosus, rheumatoid arthritis, antiphospholipid syndrome, Sjögren’s syndrome, vasculitis, and spondyloarthritis, among others. The sixth category focuses on the *‘Postpartum Period - Breastfeeding’*, covering postpartum exacerbation, drug safety during breastfeeding, and postpartum treatment approaches. The seventh category is *‘Patient Education - Counseling’*; within this framework, patient information, frequently asked questions, lifestyle recommendations, and psychosocial support are examined. The eighth category focuses on *‘Guidelines - Evidence-Based Information*’, where EULAR/ACR guidelines, scientific evidence, and other recommendations are assessed. The ninth category includes *‘Misconceptions - Risk Communication’*; incomplete or inaccurate information and narratives that can create fear are analyzed within this scope.

### Statistical analysis

Statistical analyses were conducted using the Statistical Package for the Social Sciences (SPSS) version 20.0 (SPSS Inc., Chicago, IL, USA). Before analysis, the Shapiro–Wilk test was used to assess the distribution of continuous variables; the test indicated that the distributions did not meet the normality assumption. Consequently, findings were presented as median (minimum–maximum), number (n), and percentage (%), using nonparametric statistical techniques. Videos were classified into three distinct quality categories: low, intermediate, and high. The Kruskal–Wallis test was used to compare these groups. Spearman’s rank correlation coefficient was applied to assess the correlations between variables. The inter-rater agreement during the video assessment process was assessed using Cohen’s kappa coefficient. A p-value below 0.05 was deemed statistically significant.

## Results

**Fig. 1:**
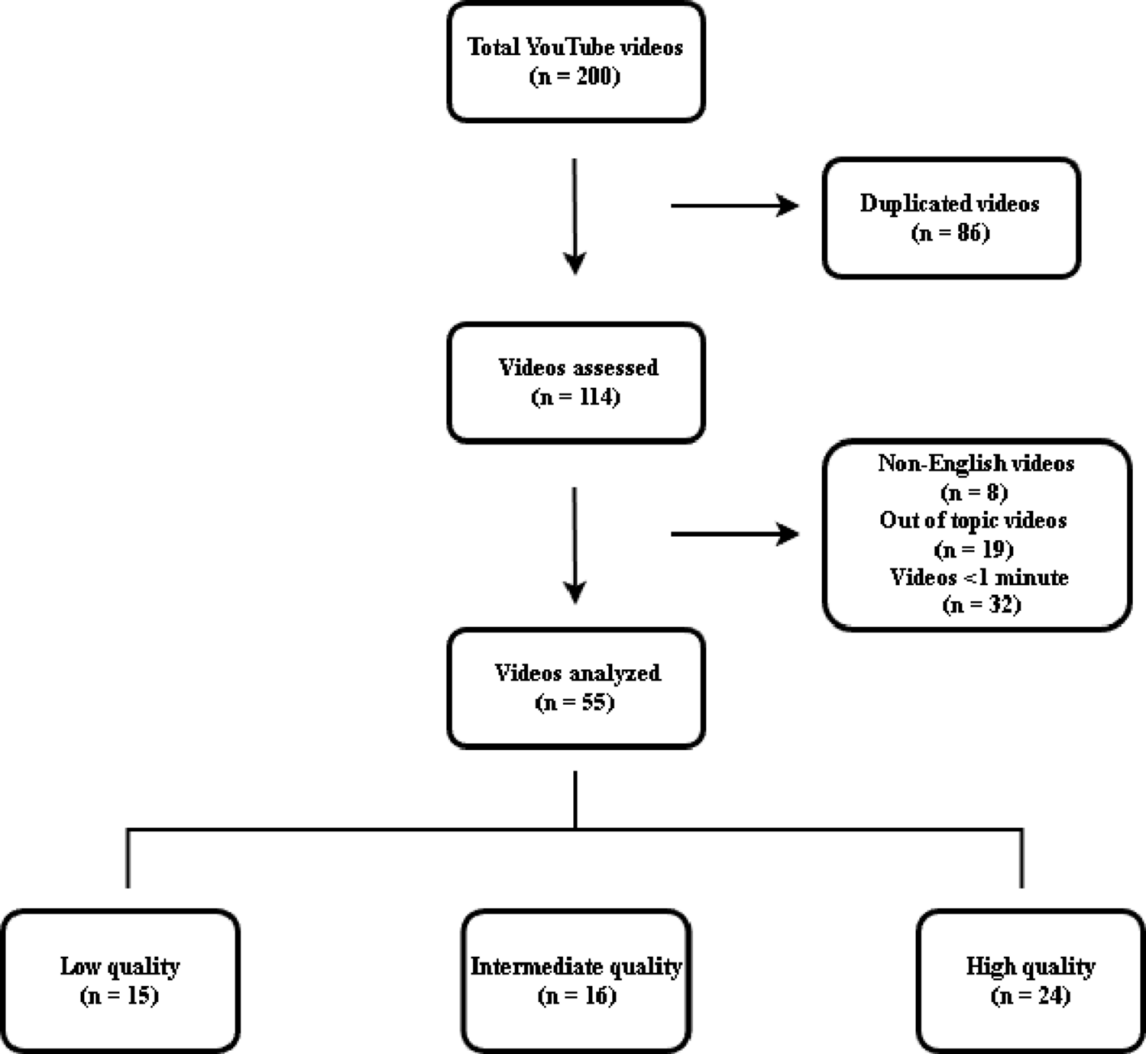
Overview of the video selection process

A total of 200 YouTube videos were initially listed. After removing duplicates, 114 videos remained for evaluation. During this process, 8 non-English videos, 19 off-topic videos, and 32 videos shorter than 1 min were excluded. Consequently, 55 videos were included in the final analysis (Fig. 1). Based on a quality assessment, 15 (27.3%) videos were classified as low quality, 16 (29.1%) as intermediate, and 24 (43.6%) as high quality.

The median duration of the videos was 286 (66-5275) seconds. The median number of views was 699 (14-38135), the median number of likes was 8 (0-377), and the median number of comments was 0 (0–72). The median time since the videos were uploaded was 1553 (35-4687) days. When the presentation methods were examined, the vast majority of the videos were in a narrative-oriented format (*n* = 35, 63.6%), followed by slide-based presentations (*n* = 6, 10.9%), patient experiences (*n* = 6, 10.9%), panel-webinar format (*n* = 5, 9.1%), and animated - visual explanatory content (*n* = 3, 5.5%) (Table 1).

**Table 1:** Primary features of the videos

Video features	Value
Duration (seconds)^*^	286 (66–5275)
Number of views^*^	699(14–38135)
Number of likes^*^	8 (0–377)
Number of comments^*^	0 (0–72)
Days since upload^*^	1553 (35–4687)
Views per day^*^	0.58 (0.06–40.27)
Likes per day^*^	0.01 (0–0.40)
Comments per day^*^	0 (0–0.07)
*Presentation method (n; %)*	
Narrative-oriented	35 (63.6)
Slide-based presentations	6 (10.9)
Animated - visual explanatory content	3 (5.5)
Patient experience	6 (10.9)
Panel - webinar format	5 (9.1)

* Data are expressed as median (minimum - maximum)

When classifying videos by source, the nonprofit charities or foundations category had the largest number of videos (*n* = 19). Among these, 13 (68.4%) were deemed high quality, 4 (21.1%) intermediate, and 2 (10.5%) low quality. Of the 9 videos created by physicians, 4 (44.4%) were high quality, 4 (44.4%) were intermediate quality, and 1 (11.1%) was low quality. For the 5 videos from academic medical centers, 3 (60%) were high quality and 2 (40%) were intermediate. Of the 9 videos from non-academic healthcare facilities, 6 (66.7%) were classified as low quality, 2 (22.2%) as intermediate quality, and 1 (11.1%) as high quality. Among the 8 videos in the TV channels / News media category, 3 (37.5%) were high quality, 3 (37.5%) were intermediate quality, and 2 (25%) were low quality. In the pharmaceutical / commercial company category, 1 (50%) of the 2 videos was low quality and 1 (50%) intermediate quality. Both videos from nonphysician healthcare professionals were classified as low quality. The single video in the independent user category was also rated low quality (Table 2).

**Table 2:** Categorization of the videos according to sources, n (%)

Source	Low quality	Intermediate quality	High quality	Total
Physician	1 (11.1)	4 (44.4)	4 (44.4)	9
Nonphysician healthcare professional	2 (100.0)	0 (0.0)	0 (0.0)	2
Academic medical centers	0 (0.0)	2 (40.0)	3 (60.0)	5
Nonacademic healthcare facilities	6 (66.7)	2 (22.2)	1 (11.1)	9
TV channels / News media	2 (25.0)	3 (37.5)	3 (37.5)	8
Nonprofit charities or foundations	2 (10.5)	4 (21.1)	13 (68.4)	19
Pharmaceutical / Commercial company	1 (50.0)	1 (50.0)	0 (0.0)	2
Independent user	1 (100.0)	0 (0.0)	0 (0.0)	1

*n* number, % percentage

Across the low, intermediate, and high quality groups, no statistically significant differences were found in daily views (*p* = 0.285) or daily comments (*p* = 0.978). A significant difference was observed in the number of daily likes, with the highest median in the low quality group (median 0.02; *p* = 0.014) (Table 3).

**Table 3:** Comparison of the video parameters between the low quality, intermediate, and high quality groups

	Low quality	Intermediate quality	High quality	*p*
Views per day	1.39 (0.06–13.02)	0.46 (0.07–5.33)	0.53 (0.07–40.27)	0.285
Likes per day	0.02 (0–0.14)	0 (0–0.03)	0.01 (0–0.4)	0.014
Comments per day	0 (0–0.02)	0 (0–0.02)	0 (0–0.07)	0.978

When the distribution of presentation methods by quality group was examined, narrative-oriented videos were in the low quality group (9; 25.7%), the intermediate quality group (11; 31.4%), and the high quality group (15; 42.9%). Slide-based presentations were classified as low quality (1; 16.7%), intermediate quality (1; 16.7%), and high quality (4; 66.6%). Animated - visual explanatory content was classified as low quality in 1 (33.3%) and intermediate quality in 2 (66.7%). Patient experience-based videos were classified as low quality (4; 66.7%) and intermediate quality (2; 33.3%). Panel- webinar format videos were classified as high quality (5; 100%) (Table 4).

**Table 4:** Distribution of presentation methods within video quality groups

Presentation method	Low quality	Intermediate quality	High quality	Total (*n*)
Narrative-oriented; n (%)	9 (25.7)	11 (31.4)	15 (42.9)	35
Slide-based presentations; n (%)	1 (16.7)	1 (16.7)	4 (66.6)	6
Animated - visual explanatory content; n (%)	1 (33.3)	2 (66.7)	0 (0)	3
Patient experience; n (%)	4 (66.7)	2 (33.3)	0 (0)	6
Panel - webinar format; n (%)	0 (0)	0 (0)	5 (100)	5

*n* number; % percentage. Percentages are calculated within presentation method categories

**Fig. 2:**
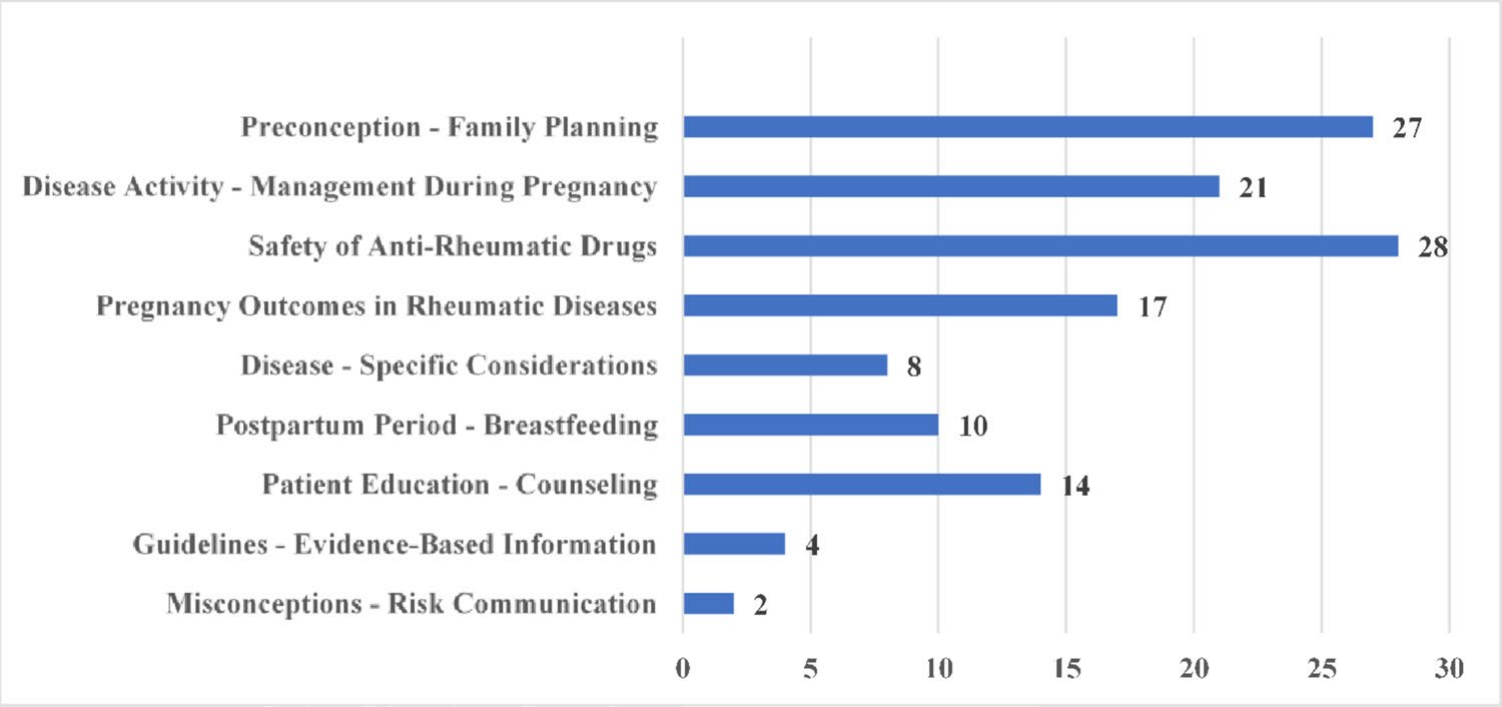
Distribution of video topics. A single video can cover multiple topic categories

The most frequently covered topic category was Safety of Anti-Rheumatic Drugs (*n* = 28), followed by Preconception - Family Planning (*n* = 27), Disease Activity - Management During Pregnancy (*n* = 21), and Pregnancy Outcomes in Rheumatic Diseases (*n* = 17). Topics that received less frequent coverage were Patient Education - Counseling (*n* = 14), Postpartum Period & Breastfeeding (*n* = 10), and Disease-Specific Considerations (*n* = 8). The categories with the least attention were Guidelines - Evidence Based Information (*n* = 4) and Misconceptions - Risk Communication (*n* = 2) (Fig. 2).

Analyses demonstrated a statistically significant positive correlation between GQS scores and modified DISCERN values (rho = 0.857, *p* < 0.001). A significant correlation was also observed between video duration and quality-assessment data (rho = 0.468, *p* < 0.001). In contrast, the duration since upload was not significantly correlated to quality scores (*p* = 0.516).

The agreement among researchers, assessed using Cohen’s kappa coefficient, was 0.81.

## Discussion

This study systematically evaluated the quality and reliability of YouTube videos related to rheumatic diseases and pregnancy. Of the 55 videos, 43.6% were high quality, 29.1% intermediate quality, and 27.3% low quality. When examining the video sources, contents from nonprofit charities or foundations and academic medical centers received higher quality ratings. A noteworthy finding was that all videos in panel/webinar format were of high quality.

Classifying 43.6% of videos as high-quality indicates that a substantial portion of contents on rheumatic diseases and pregnancy on YouTube provides an adequate level of information. The low (27.3%) and intermediate (29.1%) quality percentages suggest a mixed quality pattern of contents on the platform. Studies in the literature employing similar methodologies have reported varying rates of high-quality videos [19, 20, 21, 22], and these differences may depend on the scope of the topics, the timeliness of the video content, the search strategies, and the researchers’ evaluation approach. Furthermore, different disease groups, patient target audiences, and content creator profiles may also confound video quality.

Upon proportional evaluation, the highest quality rate was noted in the nonprofit charities or foundations category (*n* = 13, 68.4%). Likewise, videos from academic medical centers had a high quality rate of 60%, with no low-quality videos identified in this category. Accordingly, academic institutions and non-profit organizations may tend to produce more evidence-based, structured, and educationally beneficial contents [23]. In contrast, the highest rates of low quality content were observed among independent users (100%), nonphysician healthcare professionals (100%), and nonacademic healthcare facilities (66.7%). Videos from nonacademic healthcare organizations may be more promotional, contain limited scientific contents, or deviate from standard educational formats. The low quality of content produced by independent users and non-physician healthcare professionals can be attributed to their heterogeneity in expertise levels and reference practices.

The lack of significant differences between quality groups in daily views and daily comments suggests that viewing frequency or commenting behavior does not directly reflect the scientific quality of the content [24]. Notably, the higher median daily likes in the low quality group may indicate that users do not consistently evaluate content quality according to scientific criteria.

The most frequently used presentation format was narrative-oriented (*n* = 35, 63.6%). However, the quality distribution of this format was heterogeneous, with 25.7% low, 31.4% intermediate, and 42.9% high. The higher percentages of high-quality videos in slide-based presentations (66.6%) and panel/webinar videos (100%) indicate that more structured, systematic, and evidence-based content might enhance quality ratings. The incorporation of guideline summaries, statistical data, and multidisciplinary approaches in these formats may have facilitated a more comprehensive and consistent nature of the content [25]. However, the absence of high-quality patient-experience videos, with the majority (66.7%) classified as low quality, suggests that personal narratives may have limited educational depth and a weak evidence-based foundation [26]. These findings suggest that the content presentation format can be an important factor in quality assessment.

These data suggest that YouTube video providers prioritize medication safety and pregnancy planning considerations. The fact that “safety of anti-rheumatic drugs” was the most frequently addressed issue (*n* = 28) may indicate that pharmacological therapy causes the most uncertainty and anxiety for both patients and healthcare providers. DMARDs, biological agents, and drugs that must be discontinued prior to pregnancy are all important subjects in clinical practice because of concerns regarding fetal safety and teratogenicity [27]. The high frequency of discussion in the “Preconception - Family Planning” category (*n* = 27) reflects increasing awareness of the relevance of planned pregnancy in women with rheumatic diseases. Current practice guidelines emphasize core concepts, including disease activity management, pregnancy planning during remission, and the importance of a multidisciplinary approach [28]. Therefore, the content aligns with clinical priorities. On the other hand, the categories “Guidelines - Evidence Based Information” (*n* = 4) and “Misconceptions - Risk Communication” (*n* = 2) were the least covered. The limited amount of guideline-based contents may suggest that the presentation of direct evidence-based references was not sufficiently emphasized in a significant portion of the videos. Similarly, the infrequent coverage of misconceptions or risk communication indicates that the platform prioritizes information dissemination, relegating the functions of verifying information and correcting misconceptions to the background.

The strong positive correlation between GQS and modified DISCERN scores indicates that information reliability increases as content quality improves. The positive correlation between video duration and quality suggests that longer videos generally provide more comprehensive and well-structured information. Conversely, the absence of a significant correlation between video upload date and quality indicates that factors such as preparation methods and scientific foundations are more influential than the timeliness of content.

This study has several limitations. The study uses a cross-sectional approach and exclusively represents YouTube search results dated February 2, 2026. Due to the dynamic nature of YouTube contents, video availability may vary depending on the date of the search. The analysis evaluated the four specified keywords and assessed the top 50 results for each search term. This strategy, while indicative of standard user behavior, may have omitted videos that ranked lower or could be retrieved using alternative search criteria. The study considered only English videos, excluding content in other languages. This constrains the evaluation of information sources available to patient populations, especially in non-Anglophone countries. Fourthly, video quality was evaluated with the GQS, and reliability was measured using the modified DISCERN scale. Although these tools are commonly employed, the assessment process may include some subjective judgment. While Cohen’s kappa coefficient indicated high inter-researcher agreement, there remains a possibility of classification errors due to the observational nature of content evaluation. A disease-specific quantitative analysis was not conducted, which might limit understanding of how well individual conditions are represented. This study did not assess the alignment of thematic categories with patient concerns or key guideline recommendations. Finally, the study assessed fundamental interaction indicators, including views, likes, and comments; however, it did not measure outcomes such as the extent of user information acquisition, behavioral changes, or effects on clinical decision-making processes.

## Conclusion

The fact that approximately half of the videos were classified as high quality indicates that a certain level of qualified health information is available on the platform. However, the significant proportion of low and intermediate quality content suggests that information on YouTube is heterogeneous in terms of consistency and scientific accuracy. In terms of video sources, contents uploaded by academic medical centers and nonprofit charities or foundations stood out for higher quality. Regarding presentation style, the consistently high quality of all videos in panel/webinar format—and the even higher quality ratio in slide-based content—indicate that structured, systematic presentation methods can effectively enhance content quality. The most common topics in the videos were anti-rheumatic drug safety, preconception and family planning, disease activity management during pregnancy, and pregnancy outcomes in rheumatic diseases. Future research incorporating contents in non-English languages will enhance the generalizability of findings. Moreover, analyzing multiple online platforms may offer a thorough evaluation of the current. Future high-quality video contents should systematically address key clinical aspects, including preconception planning during disease remission, risk stratification by diagnosis, evidence-based recommendations on antirheumatic drugs and biologic therapies, management of clinically significant conditions, postpartum care, and references to up-to-date guidelines. Academic centers should play a leading role in producing and disseminating such structured, guideline-based contents. Developing standardized formats for educational videos may further enhance their quality, accessibility, and reliability, thereby improving patient education and supporting informed decision-making in clinical practice. Additionally, establishing a standardized format for educational videos could ensure consistency, improve understanding, and facilitate comparisons across contents. By highlighting these essential information blocks and encouraging evidence-based videos, future initiatives may enhance the real-world effectiveness of online educational resources in the field of pregnancy and rheumatic diseases.
